# Fractal analysis is a useful tool for evaluating prostate tissue remodeling caused by ethanol consumption and androgen therapy

**DOI:** 10.1590/1984-3143-AR2023-0072

**Published:** 2023-09-18

**Authors:** Bruna Jardim Pereira Lima, Gabriel Rodrigues Leal de Oliveira, Thainá Cavalleri Sousa, Ariana Musa de Aquino, Karianne Delalibera Hinokuma, Maria Luiza Silva Ricardo, Wellerson Rodrigo Scarano, Anthony César de Souza Castilho, Francis Lopes Pacagnelli, Francisco Eduardo Martinez, Leonardo de Oliveira Mendes

**Affiliations:** 1 Programa de Pós-Graduação em Ciências da Saúde, Universidade do Oeste Paulista, Presidente Prudente, SP, Brasil; 2 Programa de Pós-Graduação em Ciência Animal, Universidade do Oeste Paulista, Presidente Prudente, SP, Brasil; 3 Departmento de Biologia Estrutural e Funcional, Instituto de Biociências de Botucatu, Universidade Estadual Paulista, Botucatu, SP, Brasil

**Keywords:** chronic alcoholism, fractal dimension, prostate, testosterone

## Abstract

Alcohol has been widely consumed for centuries and is linked to the aggravation of diseases. Several studies have shown that excessive consumption of ethanol results in morphophysiological changes in the male reproductive system. One of the effects of ethanol is the decrease in testosterone concentration and hormonal therapies are an alternative to minimize the changes resulting from chronic alcoholism. Qualitative studies were commonly carried out to evaluate the male histopathological alterations resulting from ethanol consumption, being necessary quantitative and non-subjective techniques. This study analyzes the importance of fractal analysis as a useful tool to identify and quantify tissue remodeling in rats submitted to ethanol consumption and hormone therapy with testosterone. Prostate of animals submitted to chronic ethanol consumption showed tissue disorganization, which was confirmed by an increasing of fractal dimension. Regarding the prostatic stroma, collagen fractal dimension and quantification revealed lower values in animals that were only submitted to androgen therapy. Thus, we can conclude that the fractal analysis was a useful tool to quantify tissue changes caused by ethanol consumption and androgen therapy.

## Introduction

Alcohol, a psychoactive substance with addictive properties, has been widely used by society over the centuries. Alcohols’ indiscriminate use influences the development and aggravation of diseases and generates social and economic burden. Worldwide, three million deaths per year results from the harmful use of alcohol, representing 5.3% of all deaths (Paho-Who, 2020).

Due to the great epidemiological impact of alcoholism, studies are required to understanding its repercussions on the human body. Ethanol and its metabolite, acetaldehyde, cause widespread disturbances in different organ systems and act on the metabolic pathways of glucose, lipids, and proteins through the formation of reactive oxygen species, disrupting tissue homeostasis (Martinez et al., 2000; Riley et al., 2001; Gomes et al., 2002) and causing general disorders in the central nervous system, hematopoietic, reproductive and others (Fávaro and Cagnon, 2006).

Regarding the alterations in the male reproductive system reported in the literature, can be highlighted: seminiferous tubular atrophy, loss of germ cells, abnormal seminal cytology (Martinez et al., 2000), presence of gynecomastia, hypogonadism and low concentrations of testosterone, resulting in the inhibition of spermatogenesis (Galvão-Teles et al., 1986; Van Thiel et al., 1974; Gordon et al., 1976; Cicero and Badger, 1977; Van Thiel and Lester, 1979).

Androgens are steroid hormones characterized by a complex action on target-tissues such as bones, muscles, bone mass, central nervous system, and reproductive organs, including prostate. Besides, its physiological function, testosterone has become a drug widely used, especially in pathologies associated with the aging process. These pathologies are characterized by low serum levels of testosterone, including diabetes/metabolic syndrome, cardiovascular diseases, and osteoporosis (Haidar et al., 2007; Reyes-Vallejo et al., 2007).

In the scientific literature there are already several benefits linked to the use of testosterone replacement therapy (TRT) in cases of hypogonadism, which promotes gain of bone mass, decreases metabolic syndrome, and enhances lean muscle mass (Fink et al., 2018). Furthermore, TRT has been able to revert the damage caused by chronic alcoholism in the prostatic microenvironment. Mendes et al. (2014) pointed a reduction of inflammatory foci in the prostate of rats that consumed ethanol and underwent testosterone therapy (TT), as well as a reversal of the atrophy of the epithelial compartment. In addition, they also observed that hormone therapy was able to increase adhesion between epithelial cells and restore the integrity of the smooth muscle layer.

Studies involving animal models helps to elucidate the effects and mechanisms of action of ethanol. However, most of this research were carried out through the ingestion of ethanol by gavage, which does not mimic human consumption (Dantas et al., 2012; Bannach et al., 2015; Fernandes et al., 2015). Among the strain of animals that voluntarily consume ethanol, the UChB, originated from the Wistar strain after decades of crossbreeding, is the only one that keep inbreeding, and, therefore, is the only considered pure for the purposes of genetical, biochemical, physiological, nutritional, and pharmacological studies of the ethanol’ effects, as well as studies concerning appetite and tolerance, which are important factors linked to human alcoholism.

Although these authors describe the alterations resulting from the chronic consumption of ethanol in the prostatic microenvironment and others show the role of testosterone in the reversal of some parameters (Sáttolo et al., 2004; Mendes et al., 2014, 2015), the morphological analyzes used were based on qualitative characterizations, requiring studies that quantitatively and non-subjectively evaluate the histological changes observed in response to the different protocols.

The fractal analysis (FA) currently appears used in several fields of medicine. The FAis a useful parameter for the characterization of complex and irregular structures, of which analyses, examined mathematically, denote figures with self-similarity (Tambasco et al., 2009). FA detects subtle morphological changes, transforming the complexity of form into analytical quantitative data, reconciling structural characteristics and functional quantitative measures (Pajevic et al., 2018).

In research regarding prostate biology, the use of the FA as a tool for pathological diagnosis in prostate cancer has been established by several authors (Arruda et al., 2013; Waliszewski et al., 2015; Waliszewski, 2016), reinforcing the concept that the fractal estimation method is a useful additional index for conventional microscopic evaluation. Briefly, as fractal analysis can provide quantitative and reproducible information, reducing errors arising from manual and subjective analyzes and minimizing intra and interobserver variability (Frisch et al., 2012), this technique becomes a potential tool for process evaluations and pathological damage caused by drugs, including ethanol, on the tissues.

Thus, the present study aims to validate the use of fractal analysis as a tool to evaluate the effects of ethanol on the prostatic microenvironment and to perform correlation with morphological analyses.

## Methods

### Animals

UChB rats came from the vivarium of the Anatomy Department of the Biosciences Institute of the São Paulo State University - Campus of Botucatu. The animals were kept in polyethylene boxes, with 40x30x15cm dimensions, with solid bottoms, filled with wood shavings substrate, under controlled conditions of light (12h of light and 12h of dark), temperature (20 to 25°C). Filtered water and feed (Nuvital^®^) were available *ad libitum.*

### Selection of ethanol-drinking animals

After rats were born (gestation 21±1days) and weaning (rats with ±40g) at 21 days old, the rats were grouped in boxes receiving water and food *ad libitum* and, at 50 days old, the animals were individualized in separate boxes. At 65 days old, the groups of animals that received ethanol had a bottle of water *ad libitum* and another one containing a 10% ethanol solution. Both groups had their bottle positions periodically alternated to avoid conditioning. After 15 days (80 days old) of evaluating the consumption of the 10% ethanol solution, the selection and standardization of the UChB strain were perfomed (Mardones and Segovia-Riquelme, 1983). The animals that presented an average consumption between 2-6 g of ethanol/g of body weight/day were classified within the UChB strain and were destined for the experiment.

### Experimental protocol

The selected animals, at 80 days old, were divided into two experimental groups (n = 20/group): the EtOH group, which continued to receive 10% ethanol and water *ad libitum,* and the control group (C), which received only water. At 150 days old, 10 rats from each experimental group were submitted to hormone therapy, where they received subcutaneous injections of testosterone cypionate (Deposteron ® - Novaquímica, 5mg/kg of body weight) diluted in corn oil, every other day, for 30 days, always in the same period (8h00 - 8h30min). The protocol was based on the methodology used by Sáttolo et al. (2004) and Scarano et al. (2006). The animals submitted to androgen therapy constituted the experimental groups T and EtOH+T, while the other twenty animals (10 rats/group) received only corn oil as vehicle (0.2 ml/kg of body weight - groups C and EtOH).

The experimental protocol followed the ethical principles in animal research of the Brazilian College of Animal Experimentation (COBEA) and was approved by the Ethics Committee on Animal Experimentation of the IBB/UNESP (208 - CEA).

### Analysis of the prostate structure

At 180 days old, the rats were euthanized in a CO_2_ chamber followed by decapitation. The use of CO_2_ is an accepted method of euthanasia with some restrictions (in according to the recommendation of normative 37/2018 from the National Board of Control in Animal Experiments) was applied in this study due to previously published hormonal analyzes (Mendes et al., 2014, 2015) and support by the scientific literature (Deckardt et al., 2007; Nazian, 1988).

Euthanized animals were weighed and submitted to laparotomy abdomen-pelvic for removal and collection of the ventral prostate. Fragments of the intermediate segment of the ventral prostate were rapidly fixed by immersion in metacarn (6 methanol: 3 chloroform: 1 acetic acid) and maintained in 70% alcohol. After that, the material was dehydrated in increasing ethanol solutions, clarified in xylene, and embedded in paraplast (Oxford Labware, St. Louis, MO, USA).

Paraplast-embedded ventral prostate fragments were sectioned in 4 μm thick on a rotation microtome and subjected to the following stains:

1. ***Hematoxylin - Eosin (HE)***: analysis of fractal dimension and epithelial height;
2. ***Picrosirius - hematoxylin***: evidence and quantify the relative volume of collagen and analysis of the fractal dimension.

The slides were analyzed, and the microscopic fields digitized using the image analysis system (*Image Pro-Plus*) attached to the photomicroscope Leica (Leica Microsystems, Nussloch, Germany).

### Epithelial Height

The epithelium height of the prostate tissue was performed on histological sections stained with HE follows the instructions of the Image J software (National Institute of Health, United States - NIH, version 1.53t), freely available on the Internet (http://rsbweb.nih.gov/ij/). Two histological sections were analyzed per animal (5 animals/group), with an interval of 50 μm between each section, as described by Tamarindo et al. (2021).

### Quantification of the relative volume of collagen

The relative volume of collagen was quantified in picrosirius-stained histological sections following the instructions of *Image J* software (National Institute of Health, United States - NIH). Two histological sections were analyzed per animal (5 animals/group), with an interval of 50 μm between each section, and 10 histological fields/section were photographed at 40x objective, 400X magnification^,^ as described by Aquino et al. (2019).

### Fractal analysis

For analysis of fractal dimension, two histological sections/animal (interval of 50 μm between each section - 5 animals), stained with HE or Picrosirius, were digitized (10 histological fields/section, 40x objective, 400x magnification), binarized for reading (same threshold level for all images) and the fractal dimension estimated by box-counting method, using *Image J* software. The software considered the box-counting in two dimensions, allowing the quantification of the distribution of pixels in this space, not considering the texture of the image. As a result, two images with the same distribution of pixels, one binarized and the other in gray levels, had the same fractal dimension.

The analysis of fractal histological slides will be based on the relationship between the resolution and the evaluated scale, and the result is quantitatively expressed as the fractal dimension of the object, which is DF= (Log Nr / log r-1), where “Nr” is the amount of elements equals needed to fill the original object and “r” was the scale applied to the object. With this, the fractal dimension calculated with the Image J software will always be between 0 and 2, not distinguishing different textures (Cury et al., 2018).

### Statistical analysis

The results were evaluated using the statistical software Prism 8.0.1 (GraphPad), considering a confidence interval of 95%. After performing the Shapiro-Wilk normality test, non-parametric analysis was used for all parameters evaluated, using the Kruskal-Wallis test and Dunn's test a *posteriori*.

## Results

**Figure 1:**
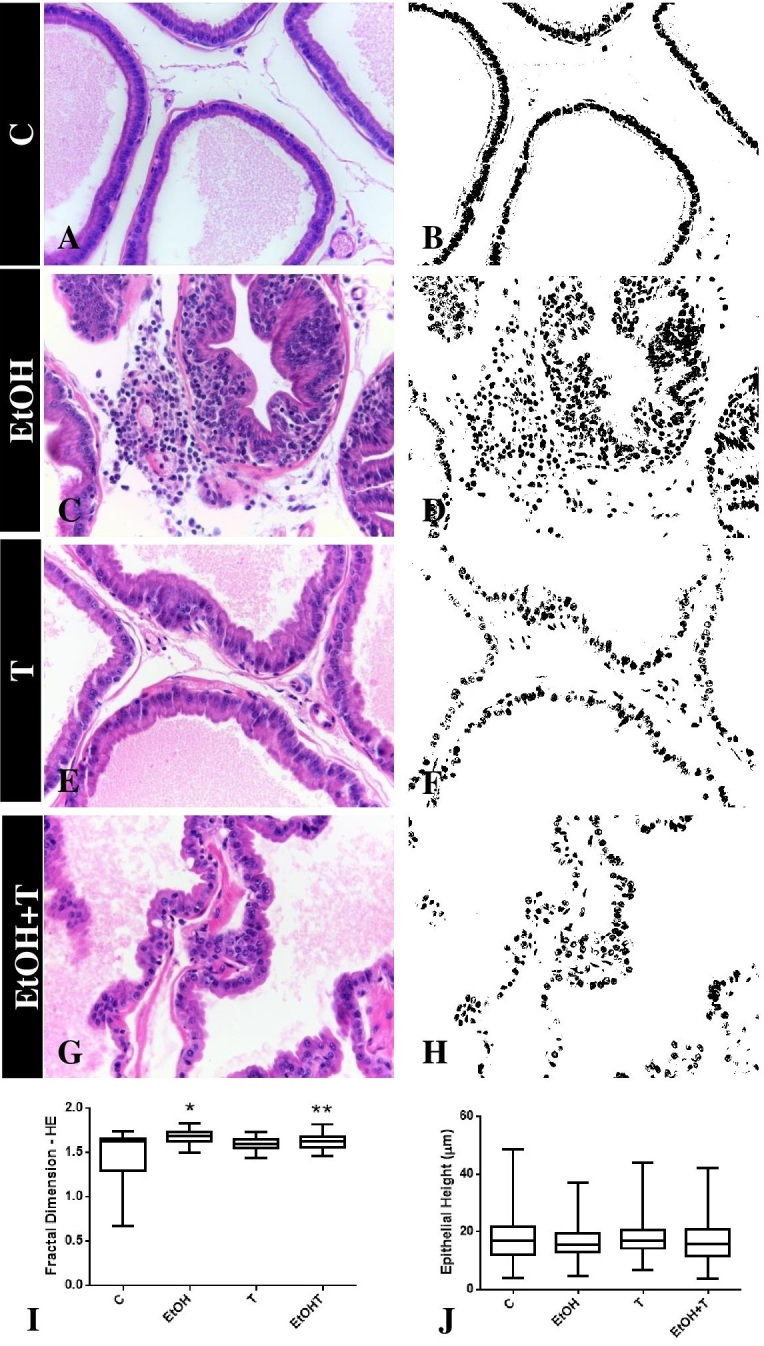
Histological sections and fractal dimension in the prostate of UChB rats submitted or not to hormone therapy with testosterone. Morphological characteristics and the corresponding image obtained after the binarization process in animals of group C (**A** and **B**), EtOH (**C** and **D**), T (**E** and **F**) and EtOHT (**G** and **H**). **Graphic rep**resentation of fractal analysis (**I**) in which there is an increase in fractal dimension in groups exposed to experimental chronic alcoholism. **Graphic rep**resentarion of epithelial height (**J**) in which there is no difference in this parameter among the groups. 400X magnification. Staining: HE Results are expressed as mean ± SEM. Kruskal-Wallis test and Dunn's test a *posteriori*. *p < 0.05 *vs* C. **p<0.05 *vs* EtOH.

After performing the fractal analysis, a higher fractal dimension was observed in the prostate of animal submitted to experimental chronic alcoholism (EtOH: 1.675±0.007 *vs* C: 1.466±0.033) (Figure 1I). The result also shows that testosterone by itself was not able to change these values (T: 1.591±0.006), but managed to reverse the effects of ethanol, similar to the control group (EtOHT: 1.620±0.008 *vs.* EtOH: 1.675±0.007) (Figure 1J). Regarding epithelial height, ethanol consumption did not change this parameter as compared to the control group (EtOH: 16.02±0.3 *vs* C: 17.392). Following the same pattern, the use of TT did not reflect changes in the height of the epithelia of the animals in the different experimental groups (Figure 1J).

The results of fractal analysis corroborate the histopathological analysis. While the control group was characterized by presenting prostatic acini with simple cubic epithelium and stroma with the presence of few cells (Figure 1A), in the EtOH group it is possible to observe intense tissue remodeling, with the presence of inflammatory foci associated with the epithelium (Figure 1D), wich is known as inflammatory reactive atypia. Changes in tissues observed in the EtOH group were not maintained after the use of TT (EtOH+T). The prostatic stroma of these animals did not show inflammatory foci and the epithelium was organized as observed in the animals of C (Figure 1G and 1H).

**Figure 2:**
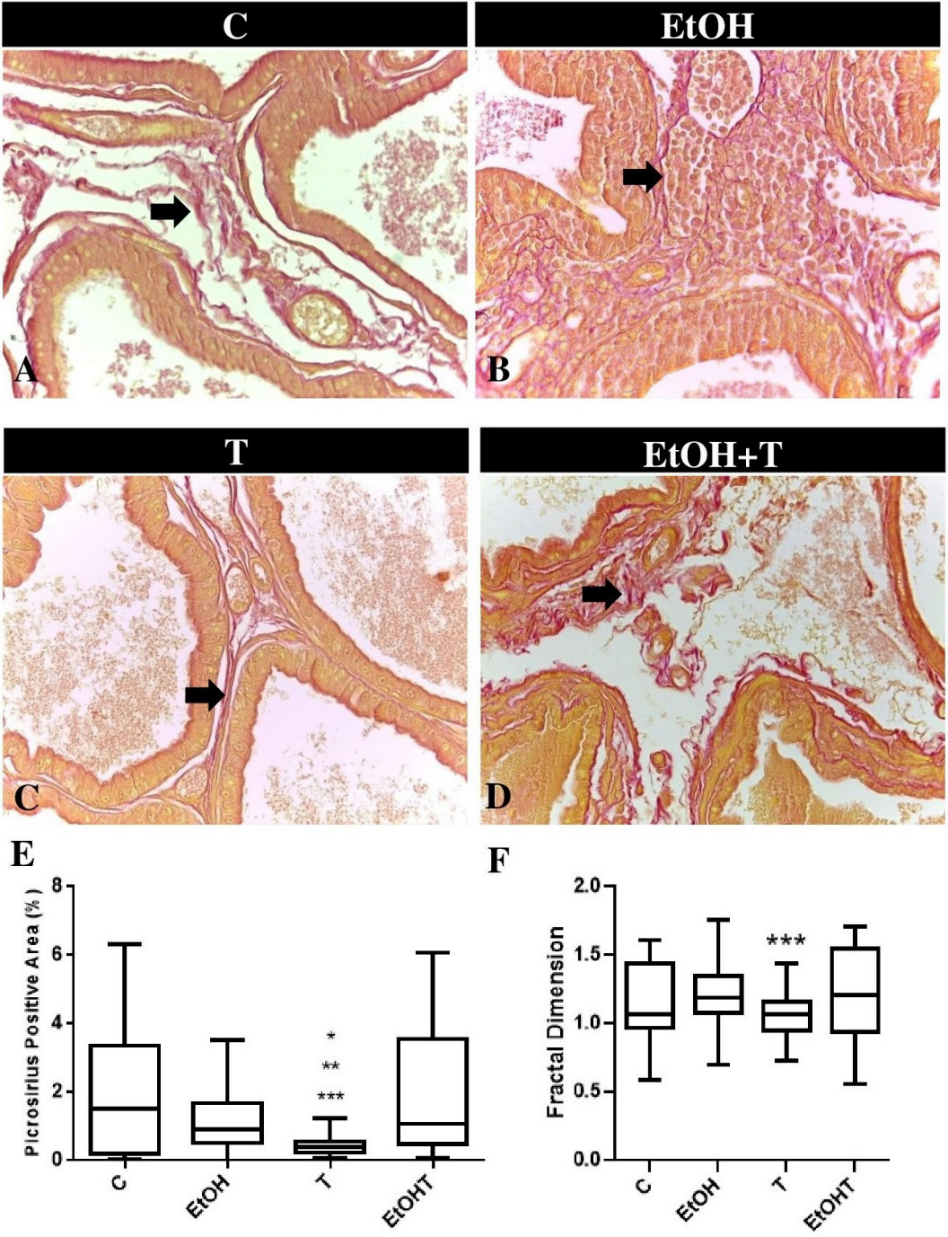
Histological sections, quantification of the area occupied by collagen fibers and fractal dimension in UChB rats submitted or not to hormone therapy with testosterone. Localization of collagen fibers (arrow) through picrosirius-red staining in animals of group C (**A**), EtOH (**B**), T (**C**) and EtOH+T (**D**). Graphics representations of the collagen quantification in the prostatic stroma (**E**) and fractal dimension (**F**). 400X magnification. Results are expressed as mean ± SEM. Kruskal-Wallis test and Dunn's test a *posteriori*. *p < 0.05 *vs* C. **p<0.05 *vs* EtOH. ***p<0.05 *vs* EtOH+T

Regarding the quantification of collagen, the results point to a reduction in the area occupied by this component in the prostatic stroma of animals submitted just to hormone therapy with testosterone (T: 0.44±0.02 vs C: 1.81±0.2) (Figures 2C and 2E).

The reduction in the area occupied by collagen in the stroma of the animals submitted to androgen therapy was accompanied by a change in the fiber organization pattern, confirmed by the reduction of the fractal dimension observed in animals of the T group (T: 1.05±0.01 vs. C: 1.15±0.03). (Figure 2F).

## Discussion

The present work demonstrates its importance in validating fractal analysis as a tool for evaluating prostatic morphological changes caused by ethanol and the role of hormone replacement with testosterone in reversing these effects. The fractal analysis agrees with the morphological techniques used in this study, indicating changes observed in the group submitted to chronic alcoholism and the reversal of this pattern after the use of TT.

In general, the increase in fractal dimension is related to the increase in tissue disorganization caused by ethanol. In the group submitted only to experimental chronic alcoholism, the presence of stromal inflammatory foci was recurrent, often associated with the epithelium, in addition to an area with atrophied epithelium. Such inflammatory characteristic attributed to ethanol was characterized by Mendes et al. (2014), who found changes in the expression of cytokines anti- and pro-inflammatory effects in the prostate of voluntary ethanol-consuming animals, as well as an increase in the number of mast cells and the expression of NFR2, a potent marker of oxidative stress. When associated with hormone therapy with testosterone, there was an absence of inflammatory foci, a fact possibly attributed to the anti-inflammatory role of testosterone (Yatkin et al., 2009; Mendes et al., 2018).

Regarding the stromal compartment, collagen fibers are the major structural components of the prostatic extracellular matrix, with type I and III collagens being the main stromal constituents (Shoulders and Raines, 2009). Research reports stromal hypertrophy with a large amount of collagen fibers associated to the presence of inflammatory infiltrates in animals exposed to experimental chronic alcoholism (Fávaro and Cagnon, 2006; Cândido et al., 2007).

The influence of ethanol on androgen levels may be the result of a direct and indirect action on the testes (Saxena et al., 1990; Tadic et al., 2000) that interferes in the hypothalamus-pituitary-testis axis (Salonen and Huhtaniemi, 1990; Tadic et al., 2000). Due to this hormonal depletion caused by the ingestion of ethanol, histological and molecular repercussions are observed in the prostate, a hormone-dependent organ. Fávaro and Cagnon. (2006) and Cândido et al. (2007) observed prostatic epithelial atrophy and widespread inflammatory foci as well as areas with pre-neoplastic lesions.

Regarding prostate tissue, several authors point to fractal analysis as a useful tool and an alternative method for diagnosing prostate cancer (Waliszewski et al., 2015; Waliszewski, 2016), but without establishing a consensus between histological and fractal analyses. While Arruda et al. (2013) establish a larger fractal dimension as the tumor aggressiveness and, consequently, tissue disorganization increase, Pu et al. (2012) reported higher values for this parameter in non-tumor tissues.

The collagen reduction detected in animals submitted to androgen therapy was also described by Felix- Patricio et al. (2017). These authors, by evaluating the prostatic stroma of orchiectomized rats, observed an increase in the collagen area and reverse results after a single dose of 100 mg /kg of body weight of testosterone decanoate. A similar mechanism was reported in an experimental model of obesity, in which decreases testosterone level presents in obese animals was associated with an increase in the collagen fibers area of the prostatic stroma (Silva et al., 2015).

Changes in collagen pattern were also related by Fávero et al. (2019) during the development of the bovine corpus luteum, in which the increase in collagen observed in the regressiona gland was accompanied by the larger fractal dimension. Greater fractal dimension was also verified in an animal model for Duchenne muscular dystrophy, linked to an increase in collagen, a different characteristic that occurred in animals that presented this pathology (Cury et al., 2018).

These authors associate tissue remodeling caused by the increase in collagen fibers to the higher expression of metalloproteinases (MMPs), such as MMP-9. These molecules are enzymes responsible for the degradation of the extracellular matrix and have a crucial role in cellular and physiological processes. Obesity, as well as hyperglycemia, another condition associated with an increase in collagen and MMPs, would be providing elements that, when cleaved, would be responsible for the release of growth factors that stimulate angiogenesis, migration, and cell proliferation^45^. According to Głowińska-Olszewska et al. (2006), abnormally high concentrations of MMP-9 may indicate changes in the metabolism of the extracellular matrix of blood vessels and muscle, these changes may be associated with atherosclerosis.

In our experimental model, the same pattern of collagen deposition associated with fibrosis was observed, with a change in this pattern after testosterone administration. This response would be interesting feature during aging process, in which the imbalance caused by the drop in testosterone concentrations is associated to the increase in fibroblasts number, smooth muscle cells and collagen fibers in the stromal microenvironment, impling an increase in glandular rigidity and the establishment of benign prostatic hyperplasia (Silva et al., 2015).

## Conclusion

The results obtained show tissue disorganization, confirmed by fractal analysis, in animals submitted to experimental chronic alcoholism. In addition, the influence of hormone therapy on the reversal of this process was detected. On the other hand, testosterone by itself was responsible for reducing the collagen fibers area, evidencing a hormonal regulation in stromal remodeling, a process also confirmed by fractal analysis. Thus, we can conclude that the fractal analysis presented itself as a reliable tool, favoring its inclusion in histopathological diagnoses.
